# Supplementary material for: The interactome of intact mitochondria by cross-linking mass spectrometry provides evidence for coexisting respiratory supercomplexes
Source: Mol Cell Proteomics. 2017 Dec 8;17(2):216–32. doi: 10.1074/mcp.RA117.000470 (PMC5795388; doi:10.1074/mcp.RA117.000470)
Supplement: Supplemental Data [file supp_RA117.000470_133922_0_supp_23978_fzffwf.zip › spectra_annotation/mito_DR_spectra_annotation/95-1-1-1-144-1.pdf]

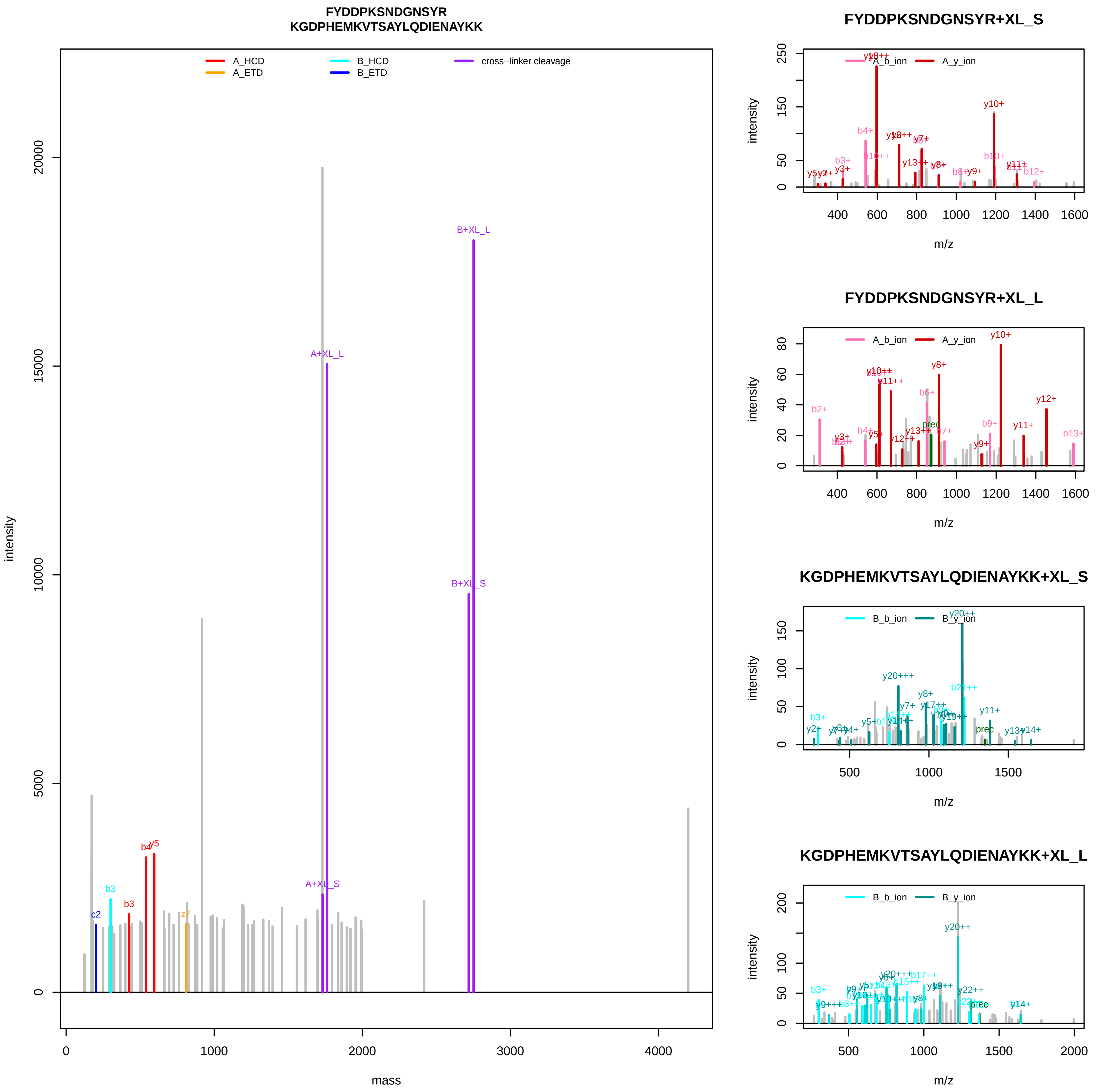
FYDDPKSNDGNSYR
KGDPHEMKVTSAYLQDIENAYKK
A_HCD
A_ETD
B_HCD
B_ETD
cross−linker cleavage
intensity
mass
B+XL_L
A+XL_L
B+XL_S
A+XL_S
c2
b3
b3
b4
y5
z7
0
5000
10000
15000
20000
0
1000
2000
3000
4000
FYDDPKSNDGNSYR+XL_S
A_b_ion
A_y_ion
intensity
m/z
y10+
y12++
y7+
y13++
y8+
y9+
y11+
b4+
b3+
y3+
b10+
b12+
FYDDPKSNDGNSYR+XL_L
y10+
y10++
y11++
y8+
b6+
y12+
y11+
y13++
y12++
y5+
b4+
y3+
b2+
b9+
y9+
b7+
b13+
prec
KGDPHEMKVTSAYLQDIENAYKK+XL_S
B_b_ion
B_y_ion
y20++
y20+++
b21++
y8+
y7+
y17++
y11+
y5+
b3+
y2+
y4+
y13+
y14+
prec
KGDPHEMKVTSAYLQDIENAYKK+XL_L
y20++
y20+++
b17++
y6+
b15++
y9++
y22++
b3+
y9+++
b8+
y10++
y13++
y8+
y14+
prec
